# Revisiting the Fight Against *Acinetobacter baumannii*: Emerging Non-Antibiotic Strategies

**DOI:** 10.3390/antibiotics15030281

**Published:** 2026-03-10

**Authors:** Victor Hugo Montini, Laura Santana Buso, Pedro Henrique Takata, Gabriel Henrique Maximino Santos, Bruna Carolina Gonçalves, Thiago Hideo Endo, Mariana Homem de Mello Santos, Eliana Carolina Vespero, Renata Katsuko Takayama Kobayashi, Gerson Nakazato

**Affiliations:** 1 Laboratory of Basic and Applied Bacteriology, Center of Biological Sciences, Department of Microbiology, State University of Londrina, Londrina P.O. Box 10.011, Brazil; laurasantana.buso@uel.br (L.S.B.); pedro.takata23@uel.br (P.H.T.); gabriel.maximino@uel.br (G.H.M.S.); bruna.carolina@uel.br (B.C.G.); th.endo@uel.br (T.H.E.); mellomariana28@uel.br (M.H.d.M.S.); kobayashirkt@uel.br (R.K.T.K.); gnakazato@uel.br (G.N.); 2 Department of Pathology, Clinical Analysis and Toxicology, Health Sciences Center, University Hospital of Londrina, State University of Londrina, Londrina P.O. Box 10.011, Brazil; elianacv@uel.br

**Keywords:** alternative strategies, antimicrobial resistance, HAIs, multi-drug resistance

## Abstract

This review discusses emerging in vitro and in vivo strategies for the control of *Acinetobacter baumannii*, a critical multidrug-resistant pathogen; the increasing isolation of strains resistant to multiple drugs, including newly developed and last-resort antibiotics, has highlighted the urgent need to pursue adjunctive therapeutic technologies. The article aims to provide an overview of alternative control approaches beyond conventional antibiotics. Emphasis is placed on strategies based on the disruption of essential metabolic pathways, nanotechnology-based approaches such as antibiotic-coated nanoparticles, in vivo bacteriophage therapy, and drug repurposing, specifically compounds such as selective serotonin reuptake inhibitors (SSRIs), as a means of exploiting already approved pharmaceuticals. By synthesizing recent findings, this review highlights current advances in the development of innovative therapeutic strategies against *A. baumannii* infections.

## 1. Introduction

The first studies involving the genus *Acinetobacter* spp. began in 1911 with the discovery of the species *Micrococcus calcoaceticus* by Martinus Beijerinck [1]. It was later renamed *Bacterium antitratum* [2], *Diplococcus mucosus* [3], *Moraxella lwoffi* var. glucidolytica [4], *M. glucidolytica* [5] and *Neisseria winogradskyi* [6]. The current genus and nomenclature only emerged after the establishment of the genus *Acinetobacter* in 1957 and after molecular analyses conducted by Bouvet and Grimont [7]. The species name “*A. baumannii*” honors the American bacteriologists Paul and Linda Baumann.

Species of the genus *Acinetobacter* are characterized as coccobacilli measuring 0.9–1.6 μm in diameter and 1.5–2.5 μm in length typically [8]. However, some studies have demonstrated that polymyxin-sensitive *A. baumannii* strains may exhibit rod-shaped morphology in the logarithmic phase, elongating in the stationary phase [9], or even assume a smaller, more coccoid shape with topographical alterations in response to desiccation and oxygen deprivation [10].

*A. baumannii* is a Gram-negative bacterium belonging to the Moraxellaceae family [11], described in 1986 by Bouvet and Grimont, who described its type-strain (ATCC 19606) as a 40–43 mol% G+C bacterium originally isolated from a urine specimen, forming circular, convex, smooth, and slightly opaque colonies capable of growth between 15 and 44 °C [7]. *A. baumannii* has gained significant clinical relevance primarily due to its involvement in healthcare-associated infections (HAIs). Its ability to tolerate desiccation, temperature fluctuations, pH variation, and nutrient-deprived conditions, as well as to form biofilms on both biotic and abiotic surfaces, combined with its rapid emergence as a global pathogen due to high antimicrobial resistance rates, renders it a critical threat to global health [10,12].

In 2021, *A. baumannii* was the second leading bacterial cause of global deaths, surpassed only by *Staphylococcus aureus*. The CRAB phenotype (Carbapenem-resistant *A. baumannii*) represented the pathogen–antibiotic combination with an increase of over 25,000 attributable annual deaths from 1990 to 2021, accounting for 78,100 deaths worldwide [13]. In fact, the World Health Organization classified CRAB as a critical pathogen for research and development of new control strategies due to its high mortality, low treatability and the unlikeliness of developing a pipeline of new drugs.

Currently, the available treatment for CRAB strains includes polymyxins, such as colistin and polymyxin B, both widely used globally despite their nephrotoxicity, as well as tetracyclines and β-lactams in combination with β-lactamase inhibitors, such as sulbactam, durlobactam, and cefiderocol [14]. Furthermore, considering the emergence of isolates resistant to the aforementioned antimicrobial [15], the use of novel strategies, particularly in combination with these antibiotics, has become an attractive approach, especially for the resensitization of these isolates.

This review aims to explore emerging strategies for combating *A. baumannii*, with a particular focus on approaches such as metabolic dysregulation, nanotechnology-based solutions, phage therapy and drug repositioning. By summarizing recent advances and highlighting ongoing challenges, this review seeks to provide a framework for the development of innovative and effective control strategies against this critical multidrug-resistant pathogen.

## 2. Can We Hijack *Acinetobacter*’s Metabolism to Defeat It?

This species demonstrates remarkable metabolic versatility and adaptability, utilizating numerous substrates as carbon sources including 2,3-butanediol, β-alanine, acetate, DL-4-aminobutyrate, DL-lactate, D-malate, ethanol, phenylacetate, glutarate, L-arginine, L-aspartate, L-phenylalanine, L-leucine, L-ornithine, L-tyrosine, malonate, trans-aconitate, and azelate [7].

A practical application of this flexibility is seen in Baumann medium, a specialized enrichment protocol where sodium acetate serves as the sole carbon source. This medium is composed of 0.2% sodium acetate (trihydrate), 0.2% KNO_3_ and 0.02% MgSO_4_·7H_2_O in 0.04 M KH_2_PO_4_–Na_2_HPO_4_ buffer (pH 6.0) and Hutner’s mineral base, under vigorous agitation [8].

This metabolic flexibility has been widely reported. Farrugia et al. [16], for instance, observed that their sequenced strains metabolized 80 out of 190 tested carbon sources, including amino acids, carboxylic acids, saccharides, and other compounds. They also noted that strains D1279779 and ATCC 17978 had a broader range of carbon and nitrogen sources compared to strains such as *A. baumannii* ACICU, suggesting a narrowing and preference in carbon utilization, particularly with an increased respiration rate of arginine, ornithine, phenylalanine, pyroglutamic acid, quinic acid, and ribonolactone.

As observed by Bouvet and Grimont [7], *A. baumannii* can utilize DL-lactate as a carbon source, which is consistent with the presence of the *dld*, *lldD*, *lldR*, and *lldP* genes, encoding D-(−)- and L-(+)-lactate dehydrogenases, a transcriptional repressor, and a lactate permease, respectively [17]. Beyond its evident nutritional role, lactate utilization, conserved in the *Acinetobacter* spp. genome, is accompanied by *lldP* upregulation during mammalian infection and enhanced survival against complement-mediated killing, compared to the mutant strain, when grown in the presence of L-(+)-lactate [17]. The dysregulation of lactate uptake and utilization, potentially achieved through irreversible competitive inhibitors identified by molecular docking approaches, may therefore represent a viable strategy for attenuating *A. baumannii*.

Leonidou et al. [18] predicted 10 genes as essential in *A. baumannii* and non-homologous to the human genome (Table 1). Examples of such genes include those associated with the shikimate pathway, involved in the synthesis of aromatic compounds such as tryptophan, phenylalanine, and tyrosine from chorismate, such as the gene encoding EPSP synthase, which converts shikimate-3-phosphate into 5-enolpyruvylshikimate-3-phosphate, and chorismate synthase. The shikimate pathway is absent from the human metabolome. Other genes include those encoding riboflavin synthase (vitamin B2) and 6-phosphogluconate dehydratase, an important enzyme in the Entner–Doudoroff pathway that catalyzes the dehydration of 6-phospho-D-gluconate into 2-keto-3-deoxy-6-phosphogluconate, a precursor of pyruvate and 3-phospho-D-glycerate. Because they lack human counterparts, these genes are promising candidates in the search for antimicrobials that inhibit, deregulate, or activate specific genes that alter bacterial fitness.

Feng et al. [19] observed that certain tricarboxylic acid (TCA) cycle intermediates, such as succinate, fumarate, and malate, as well as specific amino acids like glutamate and asparagine, can induce plasmid conjugation—such as pAB3 plasmid—through the regulation of the GacS/A system, which regulates various metabolic and physiological processes such as biofilm formation, phenylacetic acid metabolism, bacterial motility, and immune response. The impact of GacS/A on virulence itself was confirmed through analyses in *Galleria mellonella* larvae, where ∆*gacS* and ∆*gacA* mutant strains were less virulent than the wild-type and complemented strains.

In addition to GacS/A, another regulatory system, identified by Casella et al. [20], is the two-component AmsSR (Alternative Metabolic Systems), composed of a histidine kinase, AmsS, and a transcriptional regulator, AmsR. Disruption of this system is associated with the loss of proton-motive force and downregulation of the machinery responsible for both the electron transport chain and ATP synthesis. However, the decrease in ATP production via oxidative phosphorylation is compensated by diverting glucose to the glyoxylate shunt and by substrate-level phosphorylation. Other observed effects include the hyperactivation of the Pta-AckA (phosphotransacetylase–acetate kinase) pathway, which converts acetyl-CoA into acetate, as a response to prevent toxic accumulation of pyruvate.

It is also noteworthy that CRAB phenotypes also exhibit a distinct metabolic profile compared to susceptible strains, as observed by Li et al. [21], who found that CRAB strains showed reduced activity in metabolic pathways such as the TCA cycle, which leads to purine metabolism reduction and a lower proton-motive force (PMF). This led to decreased proton-motive force activity and reduced levels of AMP, ADP and ATP, as well as lower expression of enzymes associated with ADP phosphorylation. In fact, the authors observed that the meropenem-resistant phenotype could be reversed by the dose-dependent addition of AMP and ATP alongside meropenem.

The viability of CRAB biofilm cells was also reduced and in vivo tests using meropenem in combination with ATP demonstrated the effectiveness of the treatment in reducing mortality and lowering bacterial burden in the blood, spleen, liver, and kidneys in a BALB/c infection model [21].

**Figure 1:**
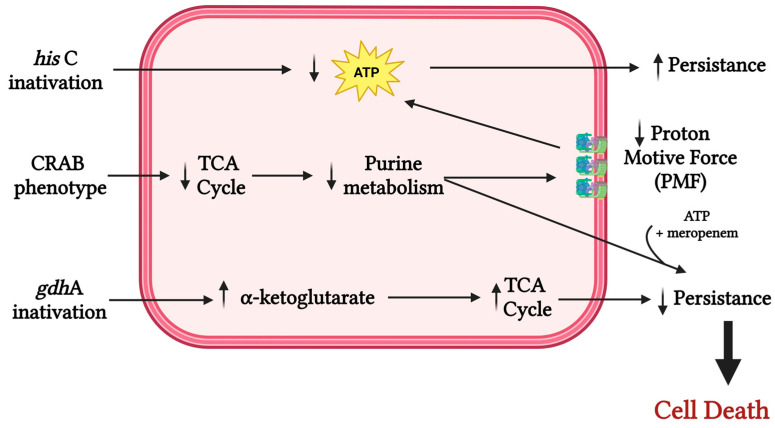
The inactivation of the *hisC* gene is associated with the histidine metabolic pathway. Its inactivation is linked to a decrease in intracellular ATP concentration, leading to increased persistence within macrophages. CRAB phenotypes exhibit reduced tricarboxylic acid (TCA) cycle activity, resulting in decreased purine metabolism, which in turn reduces the proton-motive force, lowers ATP production, and enhances persistence. This persistence phenotype can be resensitized when meropenem is added together with ATP, leading to cell death. The inactivation of the *gdhA* gene is associated with increased α-ketoglutarate concentrations, which enhances TCA cycle activity and promotes increased persistence against norfloxacin and polymyxin.

The influence of other metabolic processes on the resistance and virulence of *A. baumannii* is observed through the inactivation of the *hisC* gene, which encodes the enzyme histidinol-phosphate aminotransferase, associated with the histidine metabolic pathway [22]. Its inactivation led to an increased survival rate inside macrophages during infections, a phenomenon that can be explained by the rise in persister cells in response to low intracellular ATP levels [22]. In contrast, the inactivation of genes associated with glutamate metabolism, such as *gdhA*, which encodes a glutamate dehydrogenase, causes intracellular accumulation of α-ketoglutarate, increasing TCA cycle activity, reducing persister cell formation, and making the antimicrobials norfloxacin and colistin kinetically more effective [22] (Figure 1).

As previously mentioned, L-arginine is one of the amino acids used as a carbon source by *A. baumannii*. Deletion of the *astCADBE* operon prevents the use of arginine/ornithine as the sole carbon source [23]. Interestingly, arginine catabolism impairs bacterial fitness during infection in an in vivo murine pneumonia model, also reducing its dissemination to other organs and tissue damage [23]. The utilization of arginine during pulmonary infection is particularly important since this amino acid is present in the lungs of vertebrates and is used by the host for nitric oxide production [23].

Iron is an essential metal for bacterial metabolism and physiology, as it acts as a cofactor in enzymes, especially in cytochromes, key components of the electron transport chain, due to its ferrous (Fe^2+^) and ferric (Fe^3+^) oxidation states [24]. Although it functions as a cofactor, its involvement in numerous redox reactions facilitates the production of reactive oxygen species (ROS), particularly through its role in Fenton reactions, which are responsible for the endogenous generation of hydroxyl radicals (•OH) via interaction with H_2_O_2_ [24].

A therapeutic approach against *A. baumannii* involves iron depletion. Behm et al. [25] successfully synthesized derivatives of 4-aminoisoindoline-1,3-dione which, upon entering *A. baumannii* cells, irreversibly bind to bacterioferritin, inhibiting its interaction with its cognate ferredoxin. This leads to iron accumulation in bacterioferritin and consequent depletion of cytosolic iron. The synthesized derivatives exhibited bactericidal activity against *A. baumannii* 5075, a highly virulent and multidrug-resistant isolate, as well as synergism with colistin and an additive effect with imipenem.

In addition to the inhibition of catabolic pathways, a novel mechanism of action is the inhibition of lipopolysaccharide (LPS) synthesis. Zampaloni et al. [26] evaluated the activity of the zwitterionic benzoic acid derivative, zosurabalpin, against multidrug-resistant *A. baumannii*. The authors induced spontaneous mutations and, together with biochemical data, suggested that zosurabalpin targets the LptB_2_FGC complex, a multiprotein complex involved in LPS transport. The in vivo efficacy was confirmed in several murine models, including neutropenic pneumonia, neutropenic thigh infection, and intraperitoneally induced sepsis in immunocompetent mice, using a pan-drug-resistant (PDR) *A. baumannii* strain.

Further studies on the activity of zosurabalpin were conducted by Pahil et al. [27], who investigated the mechanism of action of three macrocyclic peptides, among which zosurabalpin was included. The authors observed that the compounds bound to the LptB_2_FG-LPS complex. Specifically, they found molecule 1 trapped the complex in an intermediate state of LPS-Lpt system, leading to a toxic accumulation of LPS. This mechanism occurs through penetration of the gate formed by the transmembrane helices of LptF and LptG, competing with the helix of LptC, a member of the Lpt complex responsible for transferring LPS from LptF to LptA. The higher activity of compound 1 and zosurabalpin, compared to compound 3 reflects the presence of bulky substituents that enable overlap with the LptC helix.

Hence, understanding the metabolism of *A. baumannii*, its responses to external stimuli and how these responses interact physiologically and genetically is also a way to design strategies that rely on unique metabolic pathways, increasing the selective toxicity and efficacy of new weapons in the war against an invisible, persistent, and constantly evolving pathogen.

## 3. When Nanoweapons Take on the Biggest Foes

Despite advances in antimicrobial discovery, *A. baumannii* remains shielded by a set of physical and functional barriers that severely limit therapeutic success. Its outer membrane, enriched with LPS, restricts drug penetration, while mature biofilms, active efflux pumps, and metabolically dormant microenvironments further reduce antibiotic efficacy. Consequently, treatment failure often reflects not the absence of active compounds, but their inability to reach effective concentrations at the site of infection. In this context, the central challenge is not merely what agent is used, but how it is delivered to the pathogen [28].

This limitation is well exemplified by tigecycline, a drug with potent in vitro activity against multidrug-resistant *A. baumannii*, whose clinical application is hampered by unfavorable pharmacokinetics and poor tissue penetration. Lan et al. [29] demonstrated that tigecycline exhibits a short in vivo half-life and limited permeability across the blood–brain barrier, preventing adequate drug concentrations in the cerebrospinal fluid despite preserved antibacterial activity. These findings exemplify a recurring theme in *A. baumannii* infections: antimicrobial potency alone is insufficient when biological barriers prevent drug accumulation at the target site. Nanotechnology emerges precisely at this interface, offering solutions to pharmacokinetic and delivery constraints rather than replacing existing antimicrobials.

Nanotechnology represents a conceptual departure from classical antimicrobial therapy by functioning not as a new drug class, but as a multifunctional platform that enhances delivery, penetration, and localized activity [30,31]. Metal nanoparticles can increase local drug concentration, interact directly with bacterial membranes, generate reactive oxygen species, and enable controlled or stimulus-responsive drug release [32]. Much like metabolic targeting and phage therapy exploit specific vulnerabilities of *A. baumannii*, nanotechnology leverages physicochemical properties such as size, surface charge, and material composition to overcome bacterial defenses.

Recent advances have also addressed concerns regarding cost, sustainability, and toxicity. Britz et al. [33] highlighted the growing relevance of green synthesis approaches, in which silver nanoparticles (AgNPs) are produced using biological reducing agents such as plant polyphenols. In their study, epigallocatechin-3-gallate (EGCG) derived from tea leaves effectively reduced silver ions into silver nanoparticles (AgNPs), improving biocompatibility while maintaining antibacterial activity. Similar environmentally friendly strategies have been reported using algae and fungi as biological factories for nanoparticle synthesis, reinforcing the notion that nanotechnology can be both scalable and sustainable [34,35].

Beyond synthesis, nanocarriers such as liposomes can also modulate the microenvironment of the infection. Hypoxia within biofilms is a well-recognized factor that compromises antibiotic efficacy. Cressey et al. [36] developed liposome-like assemblies that overcome the hypoxic limitations of biofilms, enabling effective photodynamic activity and oxidative stress generation in anoxic environments. Together, these studies frame nanotechnology not as a single intervention, but as a flexible toolbox capable of reshaping both drug behavior and bacterial physiology [31].

The biological characteristics of *A. baumannii* make it particularly susceptible to nanotechnology-based strategies. The bacterial surface carries a strong negative charge net due to LPS and other outer membrane components, favoring electrostatic interactions with cationic or functionalized nanoparticles [37]. Moreover, the integrity of the outer membrane is central to resistance against multiple antibiotic classes, meaning that physical disruption of this structure can have profound therapeutic consequences [38].

Gui et al. [39] demonstrated the relevance of surface charge manipulation by engineering positively charged imipenem-loaded zeolite imidazole-8 (ZIF-8) nanoparticles modified with polyethyleneimine (PEI) to load negatively charged imipenem. This system exploited electrostatic attractions to both retain the antibiotic and enhance interaction with the bacterial surface, resulting in improved antibacterial activity. Importantly, not all effective systems rely exclusively on electrostatic attraction. Britz et al. [33] reported that negatively charged silver nanoparticles retained strong activity against *A. baumannii* despite theoretical electrostatic repulsion, highlighting that nanoscale size and high surface area can compensate for charge-based limitations. These findings underscore that multiple physicochemical parameters, rather than a single dominant factor, govern nanoparticle–bacteria interactions.

**Figure 2:**
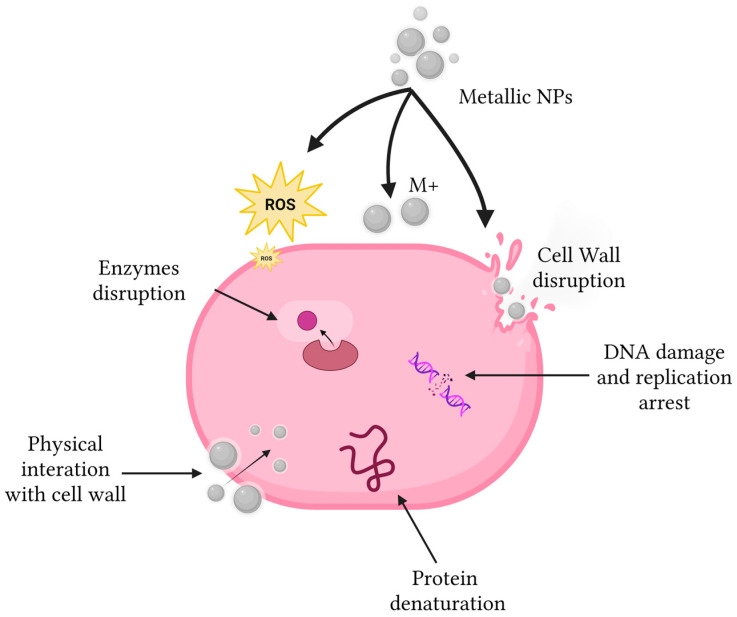
Metallic nanoparticles, particularly silver nanoparticles, exert their antimicrobial effects through the release of metal ions that can induce oxidative stress via the generation of reactive oxygen species (ROS). These nanoparticles can also interact with the cell wall, leading to its disruption and lysis, enzyme inactivation, DNA damage, and protein denaturation.

Metallic nanoparticles, particularly those based on silver, zinc, and metal–organic frameworks, have attracted considerable attention due to their pleiotropic modes of action. Unlike conventional antimicrobials that typically target a single pathway, metal-based nanostructures exert antibacterial effects through simultaneous mechanisms, including membrane disruption, reactive oxygen species generation, protein denaturation, DNA damage, and metabolic interference (Figure 2). This multimodal activity makes the emergence of resistance substantially more complex.

Gui et al. [39] showed that imipenem-loaded ZIF-8 nanoparticles eliminated *A. baumannii* by inducing oxidative stress, lipid peroxidation, and disruption of essential physiological processes. Similarly, Wei et al. [40] introduced the concept of “metalloantibiotics,” demonstrating that zinc–drug complexes form three-dimensional structures that evade efflux pump recognition, resulting in ultralow minimum inhibitory concentrations (MICs). The importance of nanoparticle size and morphology was further emphasized by Britz et al. [33], who reported enhanced antibacterial activity in particles with diameters below 10 nm, attributable to increased surface contact and interference with bacterial respiratory chains.

Beyond physical damage, metal nanoparticles can also modulate bacterial virulence. Hetta et al. [41] reported that silver nanoparticles downregulated key biofilm-associated genes such as *bap*, *ompA*, and *csuA/B*, impairing adhesion and biofilm maturation. Comparative studies indicate that while zinc oxide nanoparticles display antibacterial activity, *A. baumannii* is generally more sensitive to silver-based formulations, which may explain their predominance in experimental designs [42]. Collectively, these findings position metal nanoparticles as robust antimicrobial agents capable of exerting sustained pressure on multiple bacterial targets simultaneously.

Rather than replacing antimicrobials, nanotechnology has proven particularly effective as an adjuvant strategy that restores the activity of existing drugs. By enhancing penetration, increasing intracellular concentration, and disrupting biofilms, nanoparticles can dramatically reduce minimum inhibitory concentrations and reverse resistance phenotypes.

Gui et al. [39] quantified this effect using fractional inhibitory concentration indices, demonstrating strong synergy between imipenem and ZIF-8 nanoparticles against *A. baumannii*. Lan et al. [29] further showed that nanoformulated tigecycline achieved a 2.5-fold increase in area under the concentration–time curve and reduced systemic clearance, providing direct pharmacokinetic evidence of improved drug bioavailability. Importantly, Wei et al. [40] reported that prolonged exposure to nanoparticle-based systems over 30 bacterial generations did not select resistant mutants, contrasting sharply with the rapid resistance development observed for conventional antibiotics.

AgNPs have also been shown to resensitize *A. baumannii* to third-generation cephalosporins and last-resort drugs such as polymyxins. Allend et al. [35] observed synergistic or additive effects in all tested strains when biogenic silver nanoparticles were combined with polymyxin B, suggesting that nanotechnology may enable dose reduction in nephrotoxic agents while preserving efficacy. These findings collectively reinforce the role of nanoparticles as powerful modulators of antibiotic performance rather than standalone antimicrobials.

Biofilm formation represents one of the most formidable obstacles in the treatment of *A. baumannii* infections, conferring up to a thousand-fold increase in resistance compared to planktonic cells. The extracellular polymeric substance matrix limits antibiotic penetration and creates metabolic gradients that favor bacterial persistence. Nanoparticles, by virtue of their nanoscale dimensions, can penetrate this matrix and deliver antimicrobial agents directly to embedded cells [39,40].

Gui et al. [39] demonstrated that imipenem–ZIF-8 nanoparticles inhibited biofilm formation by more than 70%, far exceeding the activity of free imipenem. Advanced combinatorial approaches have further enhanced antibiofilm efficacy. Wei et al. [40] combined metal-based nanoparticles with photodynamic therapy, achieving near-complete eradication of mature biofilms. Xie et al. [31] emphasized that nanoparticle penetration into the extracellular matrix increases local drug concentration and uniform distribution, overcoming one of the fundamental limitations of conventional therapy.

Beyond treatment, nanotechnology also offers preventive applications. Kakian et al. [42] showed that silver and zinc oxide nanoparticles effectively eliminated biofilm-forming *A. baumannii* from inanimate hospital surfaces, highlighting their potential role in infection control; furthermore, Selim et al. [34] demonstrated in vivo efficacy, where zinc oxide nanoparticles not only cleared infection in burn wounds but also accelerated tissue regeneration. However, caution is warranted; McNeilly et al. [43,44] reported that sublethal exposure to AgNPs can select strains with enhanced extracellular polymeric substance production, resulting in denser and more resilient biofilms. These findings underscore the importance of appropriate dosing and formulation to avoid unintended selection pressures.

The most sophisticated nanotechnological strategies extend beyond passive delivery to incorporate targeting and stimulus-responsive behavior. Infection sites often exhibit acidic microenvironments due to anaerobic fermentation, a feature that can be exploited for controlled drug release. Gui et al. [39] described pH-responsive ZIF-8 systems in which acidic conditions trigger ligand protonation and antibiotic release, ensuring spatial specificity.

Lan et al. [29] exemplified dual-targeting strategies by functionalizing nanoparticles with peptides and surfactants that facilitate blood–brain barrier penetration while inhibiting efflux mechanisms, enabling effective delivery of tigecycline to the central nervous system. Even more advanced approaches integrate genetic engineering. Wei et al. [40] developed a nanoparticle composed of Zn(Bq)_2_, a metal complex, and the photosensitizer chlorin e6 (Ce6), incorporated into the ZIF-8 carrier and coated with genetically modified outer membrane vesicles that selectively recognize *A. baumannii* and achieve exceptional killing efficiency while minimizing off-target effects.

Emerging concepts such as nanozymes further expand the functional repertoire of nanotherapeutics. These materials possess intrinsic enzyme-like activity, continuously generating reactive oxygen species or degrading extracellular DNA to destabilize biofilms [45]. Such systems blur the distinction between carrier and active agent, representing a new frontier in antimicrobial design.

Despite their promise, nanotechnological approaches face significant challenges that must be addressed before widespread clinical adoption. Toxicity, biodistribution, tissue accumulation, and scalability remain key concerns. Encouragingly, several in vivo studies report minimal organ toxicity and low hemolytic activity for well-designed nanoparticle systems [29,39]. Importantly, nanotoxicity is not intrinsic but strongly influenced by modifiable parameters such as size, surface charge, and functionalization [46].

Resistance to metal-based nanoparticles, although less common, has been documented. Genetic determinants such as the *sil* operon encode efflux and sequestration mechanisms that reduce intracellular silver accumulation [46]. Additionally, correlations between high MIC values and the prevalence of efflux pump genes like *abeM* and *adeA* suggest overlapping resistance mechanisms [41]. Moreover, *A. baumannii* can develop intrinsic resistance through point mutations affecting capsule synthesis and pili formation, enhancing surface defenses and oxidative stress management without acquiring external resistance genes [43]. These findings highlight the adaptive capacity of this pathogen and reinforce the need for rational nanoparticle design and combination strategies.

With regard to guidance documents and regulatory frameworks, in 2014, the Food and Drug Administration (FDA) issued the guidance document for industry “Considering Whether an FDA-Regulated Product Involves the Application of Nanotechnology,” which outlines how the agency evaluates the use of nanotechnology in products under its jurisdiction. The document is non-binding and provides recommendations rather than legally enforceable requirements. The FDA applies two primary considerations: (1) whether the product is engineered to have dimensions at the nanoscale (approximately 1–100 nm), and (2) whether it exhibits physical, chemical, or biological properties attributable to its size, even when those dimensions extend up to 1000 nm, provided such characteristics are relevant to safety, efficacy, or performance. These considerations also extend to manufacturing changes that may affect a product’s dimensional or functional attributes.

In 2015, Brazil joined the European NANoReg project, an international initiative aimed at strengthening regulatory frameworks in nanotechnology through scientific and technical cooperation. Brazil’s participation sought to enhance the national regulatory foundation by providing legislators with a structured set of risk assessment tools and decision-making instruments to support short- and medium-term regulatory actions. This included the systematic analysis of data and the implementation of risk assessments involving exposure evaluation, monitoring, and control measures for selected nanomaterials already incorporated into commercial products.

Furthermore, Brazil has RDC No. 751/2022, issued by the Agência Nacional de Vigilância Sanitária (ANVISA), which establishes rules for the risk classification, regulatory approval, and labeling of medical devices in the country, including devices that incorporate nanomaterials. This resolution modernizes and consolidates the Brazilian regulatory framework, providing greater legal clarity and technical rigor in the sanitary approval process, particularly regarding the assessment of safety, performance, and potential toxicological risks associated with nanomaterials.

Nanotechnology does not replace antimicrobials, phages, or metabolic interventions; rather, it amplifies their effectiveness and extends their clinical utility. By addressing fundamental delivery barriers, disrupting biofilms, and enabling targeted multimodal attacks, nanotechnological platforms integrate seamlessly with existing antimicrobial strategies. From a One Health perspective, these approaches may also reduce environmental antibiotic burden by lowering required doses and limiting selective pressure beyond clinical settings [46]. In the ongoing battle against *A. baumannii*, nanotechnology represents not a singular solution, but a transformative framework capable of reshaping how antimicrobial therapies are designed, delivered, and deployed.

## 4. The Bacterial Archenemy Steps into the Ring

This section of the review delves into the most recent experimental advancements in phage therapy against *A. baumannii*, synthesizing findings from a spectrum of research models, including in silico, in vitro, in vivo and clinical applications, to provide a comprehensive overview of the current state of this emerging field.

A critical component of modern phage characterization is genomic analysis, which became the basis for viral classification according to the International Committee on Taxonomy of Viruses (ICTV) in 2022, when comparative genomic analysis was established as the foundation for their identification [47]. In silico studies of phage genomes are also critical for ensuring safety and predicting efficacy.

In recent years, researchers have consistently sequenced the genomes of newly isolated anti-*A. baumannii* phages to screen for the absence of genes encoding toxins, antibiotic resistance, and integrases, which could mediate lysogeny and potentially transfer undesirable traits to the host bacterium [48,49,50,51,52]. This genomic scrutiny is essential for selecting strictly lytic phages suitable for therapeutic development. Furthermore, phylogenetic analysis of phage genomes has revealed the vast diversity of *A. baumannii* phages, with many belonging to novel genera. For instance, recent studies have identified new members of the *Kagunavirus* and other previously unclassified viral genera, expanding our understanding of the viral ecosystem targeting this formidable pathogen [48]. This growing library of well-characterized phages is crucial for formulating effective phage cocktails capable of overcoming the narrow host range of individual phages and the potential for bacteria to develop resistance.

The in vitro efficacy of bacteriophages against *A. baumannii* is a primary determinant of their therapeutic potential. A plethora of studies published in the last two years have demonstrated the potent bactericidal activity of newly isolated phages and phage cocktails against a wide array of clinical *A. baumannii* isolates, including multidrug-resistant (MDR), extensively drug-resistant (XDR) and PDR strains [48,49,50,51,52,53,54,55,56,57]. These studies typically involve plaque assays to confirm lytic activity, determination of the multiplicity of infection (MOI) for optimal bacterial killing, and one-step growth curves to characterize the phage’s replication kinetics.

However, there remains a clear lack of standardization across the bacteriophage research community regarding the methodologies used to determine phage host range. Currently, there is no equivalent to antibiotic tests, such as disk-diffusion or broth microdilution, to assess susceptibility in the context of phage therapy. This situation arises from the intrinsic complexity of phages: as viral particles rather than simple molecules, no single assay can fully capture the multifaceted nature of phage–host interactions. Consequently, numerous studies have sought to develop comprehensive pipelines integrating in silico, in vitro, and in vivo approaches to establish a reliable and standardized workflow for accurate host range determination and the efficacy of these phages [58,59,60].

A significant challenge in treating *A. baumannii* infections is its propensity to form biofilms, which confer increased resistance to conventional antibiotics and host immune responses. Recent in vitro research has placed a strong emphasis on the antibiofilm capabilities of bacteriophages. These studies have consistently shown that specific phages can not only prevent the formation of *A. baumannii* biofilms on various surfaces but also dismantle pre-formed biofilms [61,62,63]. The mechanisms underlying this activity are often attributed to the production of phage-encoded depolymerases that degrade the extracellular polymeric substances (EPSs) of the biofilm matrix, thereby exposing the embedded bacteria to the lytic action of the phages and other antimicrobial agents.

Furthermore, the synergy between phages and antibiotics has been a focal point of recent in vitro investigations. The combination of phages with antibiotics to which *A. baumannii* is resistant has often resulted in a synergistic effect, leading to a more significant reduction in bacterial viability than either agent alone [62,64,65]. This phenomenon, known as phage–antibiotic synergy (PAS), is a promising strategy to restore the efficacy of existing antibiotics and combat the evolution of resistance. The proposed mechanisms for PAS include phage-induced disruption of the bacterial cell wall, which enhances antibiotic penetration, and selection for phage-resistant mutants that may, in turn, exhibit increased susceptibility to antimicrobials, a concept known as an “evolutionary trade-off” [66,67].

The translation of promising in vitro results to living systems is a critical checkpoint for any new therapeutic, and in this regard, phage therapy against *A. baumannii* has shown remarkable success in recent preclinical models. These studies are crucial for understanding pharmacokinetics, safety, and efficacy in a complex biological environment. The most utilized models, *G. mellonella* and various murine infection models, have consistently validated the therapeutic potential of phages. In the *G. mellonella* model, which serves as an effective high-throughput preliminary screen, studies have repeatedly demonstrated that a single dose of a specific bacteriophage or a phage cocktail can rescue the larvae from an otherwise lethal infection by MDR *A. baumannii*. For instance, Jeon et al. (2019) showed a significant increase in the survival rate of infected larvae from nearly 0% to over 50% after treatment with their novel phage, providing a clear and rapid proof-of-concept of its in vivo bioactivity [68].

Moving to more complex mammalian systems, murine models provide a more physiologically relevant setting that mimics human infections [65,68]. A key area of success has been in treating *A. baumannii*-induced pneumonia, a common and often fatal clinical manifestation. A compelling example comes from Jeon, Park and Yong [68], who investigated the efficacy of intranasal administration of the bacteriophage Bϕ-R2096 in mice with acute pneumonia caused by an MDR strain. As a result, the phage-treated mice exhibited significantly higher survival rates compared to the control group. Furthermore, histological analysis of the lung tissue revealed a marked reduction in inflammation, alveolar damage, and bacterial infiltration in the treated mice, demonstrating that the phage therapy not only cleared the pathogen but also mitigated the pathological consequences of the infection [68].

Bacteremia, or bloodstream infection, represents another life-threatening condition where phage therapy has shown significant promise [69,70]. An emblematic case is that of the lytic phages vB_AbaM_3054 and vB_AbaM_3090, which, when administered in *A. baumannii* infection models in BALB/c mice infected with extensively drug-resistant strains, demonstrated an increase in the 72 h survival rate from only 20% (untreated sepsis control group) to 80%. Additionally, Deng et al. (2016) [70] demonstrated in experimentally induced sepsis models that *A. baumannii*-specific phages significantly controlled bacterial load in the bloodstream, lungs, liver, kidneys, and spleen, with survival ratios comparable to or superior to conventional antibiotic treatment with imipenem. These in vivo studies collectively underscore two critical points: efficacy and safety. Phage administration, whether locally via intranasal routes or systemically, effectively reduces bacterial burden and improves survival outcomes. Crucially, these studies also include rigorous safety assessments, consistently reporting no adverse effects or pathological changes in the host animals, reinforcing the high specificity of phages to their bacterial targets [69,70].

While large, randomized controlled trials are the goal, the current clinical evidence for phage therapy against *A. baumannii* is built upon meticulously documented case reports under compassionate use protocols. These cases, though limited in number, offer invaluable real-world insights into the therapy’s potential and its behavior in human patients. Recent clinical cases demonstrate the promising efficacy of bacteriophage therapy against extensively drug-resistant *A. baumannii* infections in human patients [68,69,70].

Lin et al. [71] reported a case of an 80-year-old woman with severe pneumonia caused by XDR *A. baumannii*, who had failed two months of conventional antibiotic therapy, presenting with acute respiratory disease syndrome (ARDS) and pulmonary dysfunction. Nebulized phage inhalation combined with intravenous administration of polymyxin B, amikacin, and fosfomycin resulted in measurable clinical improvements within 8 days, including complete clearance of XDR *A. baumannii* from sputum cultures, substantial absorption of bilateral pulmonary lesions, reduced pleural effusion, and improved alveolar ventilation (PaCO_2_ decreased from 51.2 to 37.4 mmHg).

Additionally, Tan et al. [72] documented an 88-year-old male patient with carbapenem-resistant *A. baumannii* lung infection and underlying COPD who received personalized nebulized phage therapy (phage Ab_SZ3) in combination with tigecycline and polymyxin E for 16 days. The patient achieved bacterial clearance from bronchoalveolar lavage fluid cultures on day 7 and demonstrated significant clinical recovery with gradual resolution of bilateral consolidations and improved lung function, with no CRAB recurrence documented during the 7-month follow-up period. Earlier, LaVergne et al. [73] treated a 77-year-old male with MDR *A. baumannii* craniectomy site infection using personalized phage therapy administered intravenously, demonstrating that phage-based approaches can penetrate systemic circulation and remain viable for therapeutic intervention in serious infections caused by PDR strains.

A biological challenge relates to the potential interactions between bacteriophages and the host immune system during treatment. As viruses, bacteriophages are susceptible to immune system activity and may be rapidly cleared from the circulation following administration [74,75]. In addition, natural exposure to environmental phages means that a significant portion of the population may already possess anti-phage antibodies prior to any therapy and therapeutic administration can further induce specific IgM, IgG, and IgA responses capable of neutralizing phages and accelerating their clearance [76,77]. These immune-mediated phenomena may result in a short half-life of bacteriophages within host tissues, leading to reduced viral concentrations and, consequently, a potential loss of therapeutic efficacy.

Nevertheless, several strategies are currently being explored to overcome this limitation, which could otherwise restrict the administration of bacteriophages in immunocompetent systems. Encapsulation of bacteriophages in liposomes, for instance, can facilitate their delivery to pathogen-infected target cells, effectively acting as a “Trojan horse” [78]. Another promising approach involves the conjugation of non-immunogenic polymers such as monomethoxy-polyethylene glycol (mPEG) to viral proteins, a strategy aimed at reducing immunogenicity and thereby prolonging phage persistence within host tissues [79,80].

Despite the overwhelmingly positive results from recent experimental studies, several challenges must be addressed to facilitate the widespread clinical adoption of phage therapy for *A. baumannii* infections. The narrow host range of individual phages necessitates the development of well-characterized phage cocktails or personalized phage therapy approaches, which can be resource-intensive. The emergence of phage-resistant *A. baumannii* is another significant concern. Research has begun to unravel the molecular mechanisms of phage resistance in this pathogen, which often involve modifications to bacterial surface structures that serve as phage receptors, such as the capsule [81,82]. Understanding these resistance mechanisms is crucial for designing strategies to mitigate their development, such as the use of phage cocktails that target different receptors.

The regulatory landscape for phage therapy is also complex and varies between countries. Establishing standardized protocols for phage production, purification, and quality control that meet regulatory requirements is essential for ensuring the safety and consistency of phage preparations.

**Figure 3:**
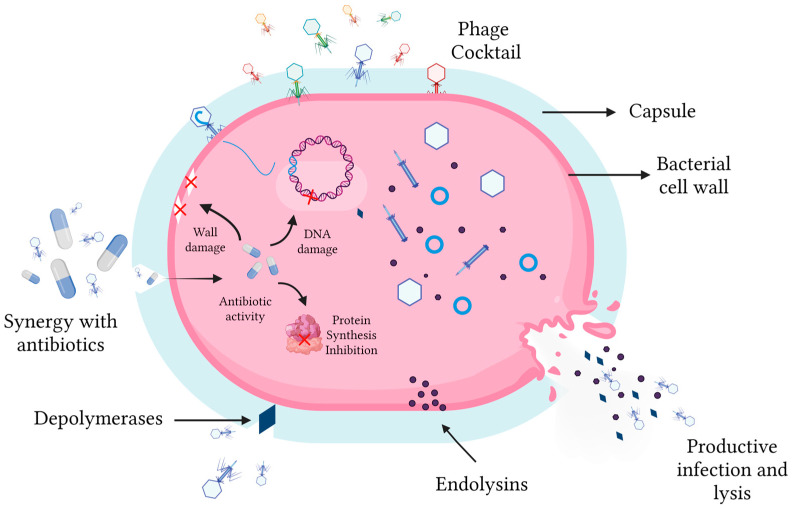
Mechanisms of action of bacteriophages that may lead to therapeutic success against *A. baumannii*. Phages can be administered as monotherapy, as part of phage cocktails, or in combination with conventional antimicrobials from various classes. In combination with antibiotics, synergism may induce damage to the cell wall, inhibition of protein synthesis, and DNA damage. These viruses can classically induce bacterial lysis through the lytic infection cycle. They may also produce enzymes such as depolymerases or endolysins, which contribute to the efficiency and success of this infection process.

Future research should continue to focus on the isolation of novel phages with broad host ranges and potent lytic activity against contemporary clinical strains of *A. baumannii*. Further in vivo studies are needed to optimize dosing regimens, routes of administration, and the timing of phage therapy in different infection models. The exploration of phage–antibiotic synergy and the use of phage-derived lytic enzymes, such as endolysins, as standalone therapeutics are also promising avenues for future investigation (Figure 3) [83].

From in silico design of safe and effective phage candidates to successful application in preclinical models and promising results in clinical case studies, the field is rapidly advancing. As our understanding of the intricate interactions between phages, bacteria, and the host immune system deepens, we are moving closer to a future where these natural predators of bacteria can be effectively harnessed to combat one of the most critical infectious disease threats of our time.

## 5. Old Drugs, New Battles: Repositioning for the War Against Antimicrobial Resistance

Drug repositioning is defined as “new therapeutic opportunities for existing and market-approved drugs” and has emerged as an increasingly studied strategy and represents a faster and more cost-effective alternative to the development of entirely new compounds [84,85,86,87,88,89]. Given the alarming rise of bacterial resistance, drug repositioning has become an appealing approach to controlling *A. baumannii* infections, offering several advantages.

The regulatory process for the development of a new drug typically takes around 15 years, whereas in drug repurposing scenarios, this timeframe can be reduced to 3–12 years [87]. Consequently, total costs are also significantly reduced, with the estimated investment for drug repositioning averaging around USD 300 million [88,89]. Another major advantage is the pre-existing knowledge of the safety profile of repurposed drugs, since pharmacokinetic, pharmacodynamic, toxicological, and clinical trial data are already available [90].

A wide range of drugs from different pharmacological classes have been investigated for potential antimicrobial repurposing (Table 2). Their mechanisms of action are diverse and include membrane permeability alteration, induction of oxidative stress, and inhibition of efflux pumps.

Several strategies have been developed to identify potential candidates for drug repositioning, which are generally classified into two main categories: experimental and in silico approaches. Experimental approaches primarily involve phenotypic screening, which consists of in vitro assays designed to evaluate the antimicrobial potential of already-known compounds. These include assays such as the determination of the MIC and minimum bactericidal concentration (MBC), as well as biofilm inhibition and eradication tests, and time–kill curve analyses, which allow the estimation of both the bactericidal activity and kinetic profile of the compound in its new therapeutic context [102,103].

Another experimental approach involves target-based interaction assays, which employ techniques such as chromatography and mass spectrometry to identify potential new biological targets for existing drugs [104].

In contrast, in silico approaches are highly diverse and rely on multiple computational tools to predict new therapeutic applications from large-scale datasets. These strategies include molecular modeling and molecular docking assays to predict the affinity between drugs and biological targets, drug–target interaction network analyses, and machine learning-based methods that integrate gene expression, transcriptomic, pharmacokinetic, and toxicological data. The use of these tools enables the prioritization of promising candidates prior to experimental validation, thereby reducing both the cost and time required for the discovery of new therapeutic indications [105,106,107]. In silico approaches have become central to antimicrobial drug repurposing by enabling the prioritization of candidates based on predicted interactions with bacterial targets before experimental validation. For example, FDA-approved drugs have been virtually screened against the *A. baumannii* resistance regulator BaeR using molecular docking, molecular dynamics simulations, binding free energy calculations, and ADMET prediction, leading to the identification of compounds with stable target interactions and favorable pharmacological profiles. Similarly, a stacking-based machine learning model was applied to screen thousands of compounds for synergistic potentiation of polymyxin E against multidrug-resistant *A. baumannii*, successfully identifying candidates that were subsequently validated through in vitro time–kill assays and murine infection models. Together, these studies exemplify how computational screening, combined with structural and pharmacokinetic prediction tools, can streamline repurposing workflows and bridge the gap between target identification and translational validation [108,109].

Several studies have demonstrated that drugs traditionally used in the treatment of chronic diseases may exhibit unexpected antimicrobial activity, either through direct mechanisms, acting on specific bacterial targets, or indirect mechanisms, such as interference with quorum sensing, biofilm formation, or the induction of oxidative stress. The most promising pharmacological classes include antidepressants, antipsychotics, antineoplastic agents, anthelmintics, and anti-inflammatory drugs [110].

Among antidepressants, selective serotonin reuptake inhibitors (SSRIs) such as sertraline and paroxetine have demonstrated antimicrobial activity against both clinical isolates and reference strains of the ESKAPE group. There is a reported MIC range from 15 to 126 μg/mL for sertraline and 60 to 250 μg/mL for paroxetine. Evidence suggests that these drugs act primarily through efflux pump inhibition, induction of oxidative stress, and disruption of bacterial membrane integrity [98]. Fluoxetine, another member of the SSRI class, exhibits antimicrobial activity against *S. aureus*, *Pseudomonas aeruginosa*, and *Escherichia coli*. Moreover, it displays synergistic effects with gentamicin and erythromycin against *P. aeruginosa* and *E. coli* [111]. The study by Foletto et al. [112] demonstrated the antibacterial activity of fluoxetine and paroxetine against multidrug-resistant clinical isolates of *A. baumannii*, *Enterobacter cloacae*, *E. coli*, *Enterococcus faecalis*, *Enterococcus faecium*, *Klebsiella pneumoniae*, and *S. aureus*, with MIC values ranging from 32 to 512 μg/mL for both drugs.

However, when considering their clinical repurposing as systemic antibacterial agents, it is important to compare these inhibitory concentrations with the plasma levels achieved under standard psychotropic dosing. At therapeutic doses (50–200 mg/day), peak plasma concentrations of SSRIs such as sertraline are typically in the range of approximately 0.1–0.2 μg/mL (≈100–200 ng/mL), which are 10- to 1000-fold lower than the MIC values reported for most bacterial species. This significant pharmacokinetic gap suggests that the direct antibacterial effect observed in vitro may not be achievable in vivo under conventional antidepressant dosing regimens.

Therefore, alternative strategies should be considered to overcome this limitation. These include the use of SSRIs as adjuvants in combination with conventional antibiotics—thereby exploiting synergistic interactions at lower, clinically attainable concentrations—as well as the development of topical formulations or targeted drug delivery systems designed to achieve higher local concentrations at the site of infection. Although systemic antibacterial use may be limited by pharmacokinetic constraints, these approaches support the continued investigation of SSRIs within antimicrobial repurposing frameworks.

Among heterocyclic antidepressants, reboxetine, a selective norepinephrine reuptake inhibitor (sNRI), has also shown promising potential for drug repurposing.

Phenothiazine antipsychotics, such as promazine and chlorpromazine, are primarily used to treat psychotic disorders, including bipolar disorder and schizophrenia. These drugs have demonstrated antimicrobial activity against *S. aureus*, *P. aeruginosa*, *E. coli*, extended-spectrum β-lactamase (ESBL)-producing strains, *A. baumannii*, and *K. pneumoniae*. Literature reports indicate that chlorpromazine can inhibit efflux pumps; moreover, it may serve as an adjuvant to conventional antibiotics, restoring their effectiveness [113]. One study showed that this drug can reduce quinolone resistance by inhibiting the plasmid-mediated OqxAB efflux pump in *E. coli* isolates from urinary tract infections, thereby enhancing antibiotic efficacy [112]. Penfluridol, another antipsychotic with antibacterial and antibiofilm properties, has demonstrated efficacy against *K. pneumoniae*, *A. baumannii*, and *P. aeruginosa*. The combination of penfluridol with colistin, a last-resort antibiotic, significantly reduced colistin MICs (by 4- to 128-fold), increased bacterial membrane permeability, and prevented the development of colistin resistance in 30-day assays [114].

Farnesol, a compound originally recognized for its anti-inflammatory properties and cosmetic applications, represents a recent example of drug repurposing with antimicrobial potential. A study demonstrated that farnesol exhibits both antibiofilm and antibacterial activity against *A. baumannii*, including multidrug-resistant isolates. Treatment with the compound was able to inhibit biofilm formation, promote detachment of mature biofilms, and reduce bacterial viability in vivo and in an ex vivo human burned skin model. The authors suggest that farnesol’s action is associated with altered membrane permeability and interference with bacterial quorum sensing. These findings highlight farnesol as a promising candidate for the development of adjuvant therapies to control persistent *A. baumannii* infections [115].

The potential of these pharmacological classes has been further reinforced by recent screening and mechanistic studies. One study identified fendiline, an FDA-approved calcium channel blocker, as a compound capable of inhibiting the growth of carbapenem-resistant *A. baumannii* strains, including OXA-23 β-lactamase-producing isolates. The study demonstrated that fendiline interferes with the lipoprotein transport system (Lol pathway), which is essential for maintaining outer membrane integrity, thereby compromising the protective barrier characteristic of Gram-negative bacteria. Furthermore, fendiline exhibited synergistic effects with β-lactam antibiotics and polymyxins, significantly reducing their MIC and restoring the susceptibility of multidrug-resistant strains [116]. These findings underscore that the rational repurposing of approved drugs represents a viable and high-impact strategy to identify agents with novel antimicrobial mechanisms, capable of overcoming bacterial resistance and expanding the therapeutic options available against critical Gram-negative pathogens such as *A. baumannii* (Table 3).

## 6. Conclusions

Through omics-based approaches, a deeper understanding of *A. baumannii* metabolism enables the exploitation of specific metabolic pathways that are absent in humans, such as the Entner–Doudoroff pathway. Targeted disruption of these pathways represents a promising strategy for the development of therapies with low toxicity and high selectivity. However, the lack of detailed studies on how *A. baumannii*’s genetic variability affects the efficacy of these disruptors remains a major limitation for the widespread application of these platforms.

In the context of nanotechnology, the functionalization of nanostructures and nanocomposites allows the incorporation of multiple properties, including antibacterial activity associated with biofilm disruption, a key determinant of bacterial persistence. When compared to non-functionalized controls (e.g., non-encapsulated nanoparticles), these approaches enable dose reduction without compromising therapeutic efficacy. However, the toxicity of many nanoparticles and their potential for bioaccumulation in the body is considered a major concern, particularly due to the lack of long-term studies.

Phage therapy has demonstrated effectiveness even in preclinical models. Due to the high viral specificity toward bacteria, this approach has become a promising strategy for infection control, particularly when combined with antibiotic therapy, promoting resensitization to conventional drugs. Although phage therapy is widely regarded as a selective and low-toxicity alternative, its broader clinical implementation remains constrained by factors such as the high host specificity of phages, regulatory bureaucracy, and the lack of standardized methodologies.

Regarding drug repurposing, it is important to highlight that the use of approved drugs with well-established pharmacokinetic profiles and known clinical effects facilitates their repositioning as antimicrobials, mainly by reducing the time and costs required for regulatory approval. It is important to highlight the gap between the in vitro antibacterial concentration and therapeutic concentration, as well as the potential side effects of these drugs, which may restrict their use, particularly in individuals who are sensitive or predisposed to adverse reactions.

Despite the potential of these strategies, there remains a critical need for robust studies evaluating therapeutic effectiveness and toxicity in in vivo and clinical models, as well as interactions between antimicrobial agents and the immune system. Even when promising in preclinical settings, the transition from experimental models to clinical application remains a major bottleneck. The absence of consolidated regulatory frameworks for approaches such as nanotechnology and phage therapy in many countries represents a significant barrier, reinforcing the need to establish clear regulatory structures, methodological standardization, and alignment between scientific development, industrial feasibility, and regulatory requirements. Furthermore, from a translational perspective, assessments of commercial viability, scalability, and manufacturing feasibility are essential. Moreover, the persistent ‘valley of death’, the funding gap that often prevents promising early-phase clinical trials from progressing to later-stage development, continues to impede the advancement of innovative antimicrobials.

Taken together, these approaches represent a rapidly expanding research field, reflecting ongoing efforts to develop novel therapeutic strategies in a post-antibiotic era. Finally, although several methods have demonstrated in vitro and in vivo efficacy, the need for experimentation in controlled clinical environments still limits the large-scale application of these compounds or agents.

## Figures and Tables

**Table 1:** *A. baumannii* genes absent in humans. Adapted from Leonidou et al. (2024) [18].

Gene ID	Gene Name	Protein Name	EC Numbers
A1S_0223	*ribE*	riboflavin synthase alpha chain	2.5.1.9
A1S_0413	*ptsP*	phosphoenolpyruvate-protein phosphotransferase	2.7.3.9
A1S_0483	*edd*	phosphogluconate dehydratase	4.2.1.12
A1S_0484	*eda*	hypothetical protein, 2-dehydro-3-deoxyphosphogluconate aldolase	4.1.2.14 4.1.3.42
A1S_0545	*ilvC*	acetohydroxy acid isomeroreductase	1.1.1.86
A1S_0585	*panB*	3-methyl-2-oxobutanoate hydroxymethyl transferase	2.1.2.11
A1S_1694	*aroC*	chorismate synthase	4.2.3.5
A1S_2276	*aroA*	hypothetical protein, EPSP Synthase	1.3.1.43 1.3.1.12 2.5.1.19
A1S_2530	*pabC*	4-amino-4-deoxychorismate lyase	4.1.3.38
A1S_2697	*cysG*	multifunctional protein	2.1.1.107 1.3.1.76 4.99.1.4

**Table 2:** Drugs repurposed as antimicrobials and their mechanisms of action.

Drug	Original Use	Mechanisms of Action	Ref.
Auranofin	Anti-rheumatic	Induction of oxidative stress	[91]
Niclosamide	Anti-helminthic	Quorum sensing suppression, biofilm formation inhibition, alteration of cell membrane charge	[92]
Ciclopirox	Antifungal	Alterations in the cell wall	[93]
Ebselen	Antioxidant	Induction of oxidative stress	[94]
Disulfiram	Alcoholism treatment	Iron chelation	[95]
Tamoxifen	Antineoplastic	Alterations in membrane permeability	[96]
Sertraline	Antidepressant (SSRI)	Alterations in the cell wall and membrane permeability, oxidative stress induction, efflux pump inhibition	[97,98]
Chlorpromazine	Antipsychotic	Oxidative stress, membrane permeability alteration, and DNA damage	[99]
Pentamidine	Antiprotozoal	Alterations in membrane permeability	[100]
Simvastatin	Hypolipidemic	Biofilm formation inhibition and efflux pump inhibition	[101]

**Table 3:** Comparative assessment of alternative antimicrobial strategies.

Strategy	In Vivo Effectiveness	Ease of Regulatory Approval	Implementation Cost	Key Limitations
Metabolic disruptors	From low to moderate (often used to resensitize antibiotics)	Moderate, regulatory, classified as antibiotics in case of antibacterial molecules such as zosurabalpin	High	Genetic variability, potential bacterial metabolic bypass; complex platforms such as interference RNAs and CRISPR-based systems may face manufacturing and scalability challenges
Bacteriophages	From moderate to high, whether with isolated phages, in cocktails, or in combination with antibiotics.	None overall (with the exception of some countries)	Low	High specificity of phages, bureaucracy, and lack of standardization of techniques.
Nanotechnology	From moderate to high, requiring combination with a bioactive compound, such as metals or another antimicrobia	Low	Low to moderate (depending on the composition of the nanocomposite).	Toxicity, metal bioaccumulation and unsatisfactory drug release
Drug repositioning	Low as monotherapy; moderate as adjuvant	High (existing safety data available)	Low	Pharmacokinetic gap between antimicrobial MIC and therapeutic plasma levels

## Data Availability

No new data were created or analyzed in this study. Data sharing is not applicable to this article.
